# Chronic alcohol consumption accelerates cardiovascular aging and decreases cardiovascular reserve capacity

**DOI:** 10.1007/s11357-025-01613-w

**Published:** 2025-03-20

**Authors:** Partha Mukhopadhyay, Burhan Yokus, Bruno Paes-Leme, Sándor Bátkai, Zoltán Ungvári, György Haskó, Pal Pacher

**Affiliations:** 1 Laboratory of Cardiovascular Physiology and Tissue Injury, National Institute on Alcohol Abuse and Alcoholism, National Institutes of Health, Bethesda, MD USA; 2 Vascular Cognitive Impairment, Neurodegeneration, and Healthy Brain Aging Program, Department of Neurosurgery, University of Oklahoma Health Sciences Center, Oklahoma City, OK USA; 3 Oklahoma Center for Geroscience and Healthy Brain Aging, University of Oklahoma Health Sciences Center, Oklahoma City, OK USA; 4 Department of Anesthesiology, Columbia University, New York, NY USA

**Keywords:** Alcohol, Cardiac function, Vascular function, Aging, Senescence

## Abstract

The pathology of cardiovascular aging is complex, involving mitochondrial dysfunction, oxidative and nitrative stress, oxidative DNA injury, impaired lipid metabolism, cell death, senescence, and chronic inflammation. These processes lead to remodeling and structural changes in the cardiovascular system, resulting in a progressive decline in cardiovascular reserve capacity and health, and an increased risk of diseases and mortality. Excessive alcohol consumption exacerbates these risks by promoting hypertension, stroke, arrhythmias, coronary artery disease, cardiomyopathy, and sudden cardiac death, yet the effects of chronic alcohol consumption on cardiovascular aging remain unclear. Herein, we explored the impact of a 6-month 5% Lieber-DeCarli alcohol diet in young (3 months old) and aging (24–26 months old) Fisher F344BNF1 rats. We assessed detailed hemodynamics, mitochondrial function, oxidative/nitrative stress, lipid metabolism, inflammation, cell death, senescence, and myocardial fibrosis using the pressure–volume system, isolated vascular rings, and various histological, biochemical, and molecular biology methods. Alcohol consumption in both young and aging rats impaired mitochondrial function, disrupted cholesterol and triglyceride metabolism, and increased oxidative/nitrative stress, inflammation, cell death, and senescence, leading to a decline in systolic contractile function. In aging rats, alcohol further exacerbated diastolic dysfunction and myocardial fibrosis. Alcohol also increased oxidative/nitrative stress, apoptosis, and senescence in the vasculature, contributing to endothelial dysfunction and increased total peripheral resistance. Additionally, alcohol exacerbated the aging-related ventriculo-arterial uncoupling and diminished cardiac efficiency, further reducing cardiovascular reserve capacity. In conclusion, chronic alcohol consumption promotes cardiovascular aging and further diminishes the already impaired cardiac and vascular reserve capacity associated with aging.

**Supplementary Information:**

The online version contains supplementary material available at 10.1007/s11357-025-01613-w.

## Introduction

Cardiovascular aging is a multifaceted process shaped by numerous interconnected mechanisms, including mitochondrial dysfunction, oxidative and nitrative stress, oxidative DNA injury, poly(ADP-ribose) polymerase and apoptotic cell death, cellular senescence, impaired lipid metabolism, and chronic inflammation, to name a few [1–10]. Recent studies also highlight the importance of interorgan crosstalk (e.g., the gut-liver-heart axis) in the development of cardiovascular dysfunction associated with cardiovascular aging [5, 11, 12]. Because of these pathological processes aging leads to various physiological and structural changes in the cardiovascular system, progressive decline in cardiovascular health, increasing the risk of cardiovascular diseases and mortality [8, 9, 13–15].

Numerous recent genetic studies on humans suggest that alcohol may significantly impact genetic aging and lifespan, with effects varying by dose and duration [16–21]. Higher doses of alcohol consumption appear to accelerate genetic aging and potentially shorten lifespan [16–21]. This is particularly alarming given the rising alcohol use among adults aged 65 and older, a rapidly expanding demographic [22]. This trend raises significant concerns due to the increased risks of cognitive decline, injury and death from falls and other accidents, and higher incidences of cancer, sleep disturbances, liver disease, and cardiovascular disease [22].

Excessive alcohol consumption heightens the cardiovascular risks by causing hypertension, stroke, arrhythmias, coronary artery disease, cardiomyopathy, and sudden cardiac death [23–26]. Despite these known dangers, the full impact of chronic alcohol consumption on cardiovascular aging remains unclear and requires further investigation.

In this study we investigated the effects of 6 months chronic alcohol consumption on detailed hemodynamic (vascular and cardiac function), cardiac mitochondrial function, oxidative and nitrative stress, lipid metabolism, inflammation, cell death, senescence, cardiovascular remodeling, and reserve capacity using young and aging Fisher F344BNF1 rats, a well-established model of cardiovascular aging.

Our findings indicate that chronic heavy alcohol consumption accelerates all the key processes in the heart and blood vessels associated with cardiovascular aging. Moreover, it exacerbates the cardiovascular uncoupling linked to aging, thereby reducing the reserve capacity of the cardiovascular system. These findings may have significant clinical implications and enhance our understanding of the adverse cardiovascular effects of chronic alcohol consumption.

## Method

### Rat chronic alcohol model

Male Fisher F344BNF1 rats were obtained from the National Institute on Aging for this study. The study was reviewed and approved by the Institutional Animal Care and Use Committee of the National Institute on Alcohol Abuse and Alcoholism (LCPTI-PP-2) and conformed to the National Institutes of Health guidelines on animal experiments (Guide for the Care and Use of Laboratory Animals prepared by the National Academy of Sciences and published by the National Institutes of Health [publication 86–23, revised 1985]). Rats were kept in a specific pathogen-free facility, with constant temperature (22 ± 2 °C), humidity and 12–12 h alternating light cycle. They received humane care and experiments were carried out during daylight conditions. Young rats (3 months old) and aging rats (24–26 months old) were divided into groups and fed either a 5% (vol/vol) liquid alcohol Lieber-DeCarli diet or an isocaloric control diet (82 Shake and Pour control liquid diet from Bio-Serv, Frenchtown, NJ, products No. F1259SP and F1258SP) for six months. ​ The Lieber-DeCarli diet is a widely used liquid nutritional regimen in experimental models of alcohol-associated liver disease (ALD) in rodents. Its primary advantage is the precise control over nutrient composition and ethanol intake, enabling detailed investigation of alcohol-induced liver pathology [27]. However, a notable limitation in pair-fed control groups is the high carbohydrate content, which may influence metabolic outcomes and complicate direct comparisons.

Each cage housed two rats with two feeding tubes provided. The liquid diet was prepared fresh daily, with intake measured and adjusted for the pair-fed control group as previously described [28, 29]. On average, the liquid diet intake was 90–110 ml per rat per day. The study included 12 young rats in each dietary group (control diet and 5% alcohol) and 20 aging rats in each dietary group. During the six-month study, four aging rats from each group (control and alcohol diet) were excluded due to the development of various age-related tumors (lymphoma, kidney, or skin tumors).

**Fig. 1:**
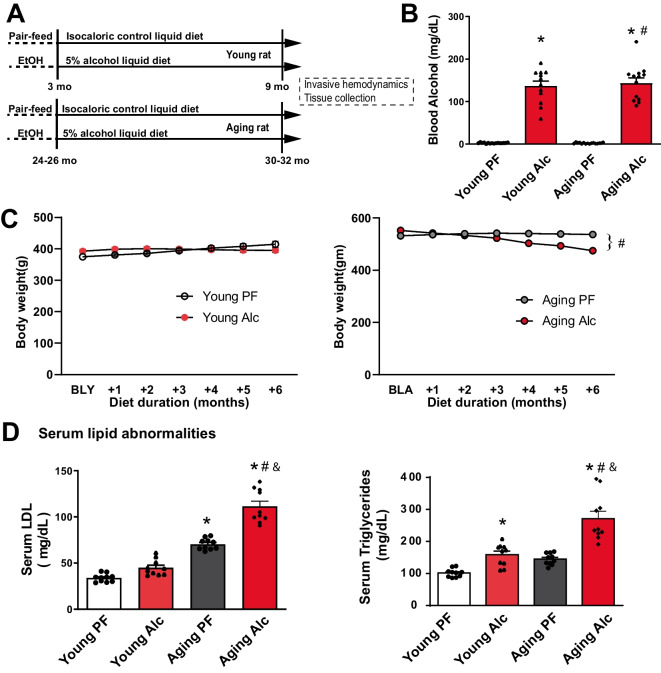
Effect of chronic alcohol liquid diet in young and aging rats on body weight, blood alcohol, and serum lipid levels. **A **Schematic diagram of the alcohol feeding protocol for 6 months. Young and aging rats were pair-fed (PF) with either an isocaloric control diet or a 5% alcohol diet (Alc). **B **Determination of blood alcohol levels from rat serum samples using an Analox machine. Data represents the mean ± S.E.M, with *n* = 12–16 per group. *p < 0.05 vs. Young PF group, # *p* < 0.05 vs. Aging PF group. **C **Changes in body weights of young and aging rats over 6 months of feeding with the indicated diets. Baseline values for young (BLY) and aging (BLA) rats were recorded at the start of the study (at 3 or 24–26 months, respectively). Data represents the mean ± S.E.M, with *n* = 12–16 per group. *p < 0.05 vs. Young or Aging PF group. **D **Serum LDL cholesterol and triglyceride levels following 6 months of dietary intervention. Data represents the mean ± S.E.M, with *n* = 8–10 per group. *p < 0.05 vs. Young PF group, # *p* < 0.05 vs. Aging PF group, & *p* < 0.05 Young Alc vs. Aging Alc group

At the end of the 6-months study period functional measurements and tissue collection were carried out (Fig. 1A). Serum samples and snap-frozen left ventricular tissue samples were stored at −80 °C for biochemical analysis. For histological examinations, formalin-fixed samples were embedded in paraffin and stored at room temperature (RT).

### Blood alcohol level

Serum alcohol levels were measured in the morning in rats by AM1 Analox Alcohol Analyzer according to the manufacturer’s instruction. While most rats begin drinking early in the morning, not all follow this pattern, leading to expected variations in alcohol levels. Given the rapid alcohol metabolism in rats, the measured levels likely represent values close to the peak concentrations achievable with this diet protocol only in some rats.

### Invasive hemodynamics and pressure–volume analysis

Hemodynamic measurements were conducted under isoflurane anesthesia (1–2%, with intubation and mechanical ventilation) as previously described [5, 30, 31]. Parameters measured/calculated included mean arterial pressure (MAP), cardiac output (CO), stroke work (SW), total peripheral resistance (TPR), the maximal slope of systolic pressure increment (dP/dt) and decrement (-dP/dt), the time constant of left ventricular (LV) pressure decay (TauWeiss), and LV end-diastolic pressure (LVEDP). The slope of the LV end-systolic pressure–volume relationship (ESPVR; quadratic model; maximal elastance (Emax) often referred to as End-Systolic Elastance (Ees)), preload recruitable stroke work (PRSW), and + dP/dtmax-end-diastolic volume relationship (+ dP/dt-EDV) were calculated as load- and heart rate-independent indices of cardiac contractility. Ventriculo-arterial coupling (VAC) was assessed by calculating the ratio of arterial elastance to end-systolic elastance (slope of ESPVR) and mechanical efficiency by ratio of stroke work (SW) and pressure–volume area [5].

### Histology, immunohistochemistry (myocardial fibrosis, 4-hydroxynonenal (4-HNE), and 3-nitrotyrosine (3-NT) staining)

Samples of rat hearts were fixed in 10% neutral buffered formalin and then embedded. The tissue blocks were sliced into 4 μm sections, which were subsequently deparaffinized and stained with Sirius Red and Masson’s Trichrome (Richard-Allan Scientific, Kalamazoo, MI). For immunostaining with F4/80, 4-HNE, and 3-NT, heart tissue sections were deparaffinized and rehydrated in descending grades of ethanol. A heat-mediated antigen retrieval procedure was then performed (pH = 6 citrate buffer or pH = 9 Tris/EDTA at 95 °C for 15 min). The sections were then incubated in BlOXALL solution (Vector Laboratories, Burlingame, CA, USA) to block endogenous peroxidase activity as per the manufacturer’s instructions. Subsequently, the sections were incubated overnight at 4 °C in a humidified chamber with anti-F4/80 antibody (1:5000 dilution; ab 300,421, Abcam, Cambridge, MA, USA); or anti-3-nitrotyrosine (1:100 dilution; #10,189,540 Cayman, Ann Arbor, MI, USA); or anti-4-HNE antibody (1/200 dilution; #MHN-100P Institute for the Control of Aging, Nikken SEIL Co, Fukuroi, Shizuoka, Japan). The next day, the sections were incubated with an anti-rabbit or anti-mouse IgG conjugated with a horseradish-peroxidase polymer (ImmPress reagents, Vector Laboratories) as per the kit’s instructions. Color development was induced by incubation with a DAB reagent (Vector Laboratories) for 30–180 s, and the sections were counter-stained with hematoxylin. Finally, the sections were dehydrated in ethanol, cleared in xylene, and mounted. For Sirius Red staining, rat heart sections were stained with Sirius Red to visualize heart fibrosis. Briefly, the sections were treated with phosphomolybdic acid (Phosphomolybdic Acid 0.2% Aqueous, Electron Microscopy Sciences, Cat #26,357–01) for 2 min and then stained in saturated picrosirius red solution (Electron Microscopy Sciences Cat #26,357–02) for 90 min. After washing with hydrochloric acid (0.01 N Hydrochloric Acid, Electron Microscopy Sciences, Cat #26,357–03), the sections were dehydrated through 3 changes of 100% ethanol and xylene and then mounted in a resinous medium. All images were captured using an Olympus BX-43 microscope set (Olympus, Center Valley, PA), and 7–10 random HPF areas were taken. The morphometric examination was performed in a blind manner [5].

### Serum lipid profiles

Serum LDL and triglycerides were measured using Cholesterol Assay Kit – LDL and Triglyceride Assay Kit (Abcam, CA, USA) according to manufacturer instructions.

### RNA isolation, reverse transcription, and Real-time PCR

Heart tissues were homogenized in Tri reagent and total RNAs were isolated with Direct-zol RNA Miniprep plus kit (Zymo Research, CA, USA). RNA concentrations were measured with a NanoPhotometer® (Implen, Westlake Village, CA, USA). 2 µg RNA was, reverse-transcribed (High-Capacity cDNA Reverse Transcription Kit, Applied Biosystems, CA, USA) and the target genes were amplified using the standard SybrGreen based real-time PCR reagent (SYBR® Select Master Mix, Applied Biosystems, Foster City, CA). Gene expression data were normalized to the expression of housekeeping genes (β−2-microglobulin and β-Actin). All primers details were previously described [5, 11] and provided in Supplementary Table 1: Primers used in this study.

### Measurement of vascular reactivity in isolated rat aortic rings

Thoracic aortae were excised, cleaned of periadventitial fat, and cut into 3- to 4-mm wide rings using an operating microscope. These rings were placed in organ baths filled with warmed (37 °C) and oxygenated (95% O2, 5% CO2) Krebs’ solution containing CaCl2 (1.6 mM), MgSO4 (1.17 mM), EDTA (0.026 mM), NaCl (130 mM), NaHCO3 (14.9 mM), KCl (4.7 mM), KH2PO4 (1.18 mM), and glucose (11 mM). Special care was taken during the preparation to avoid damaging the endothelium. Isometric tension was measured using isometric transducers (Kent Scientific Corporation, Litchfield, CT), digitized with a MacLab A/D converter, and stored and displayed on a Macintosh computer. A tension of 1.5 g was applied, and the rings were equilibrated for 60 min. After precontraction with epinephrine (10^–6^ M), relaxation responses to acetylcholine (10^–9^ to 3 × 10^–4^ M) were measured as previously described [32–34].

### Determination of myocardial caspase 3/7, PARP-1 and mitochondrial complex activities

An equal amount of protein from heart lysates was used. PARP1 (PARP1 Enzyme Activity Assay, #17–10,149, Millipore, Burlington, MA, USA), caspase 3 (Caspase-3 Assay Kit, #AB39383, Abcam) and mitochondrial complex I, II, IV activities (Complex activity assay kits, #AB109721, #AB109908, #AB109911, Abcam, CA, USA) were assessed according to the manufacturer’s instructions as previously described [5]. Results were expressed as fold change to the young control group.

### Oxidative stress markers nitrotyrosine (NT) and 4-hydroxynonenal (HNE) content

Quantitative determination of nitrotyrosine in heart tissues were determined using Nitrotyrosine ELISA kit (Hycult Biotecch Inc, PA, USA) [5]. Quantitative determination of 4-HNE adducts in protein was measured by 4-Hydroxynonenal ELISA Kit (Abcam, CA, USA).

### Vascular ROS measurements

Hydrogen peroxide production was measured fluorometrically in aortic segments using the Amplex red/horseradish peroxidase assay. Data were normalized to tissue weights and reported as relative differences in H2O2 production (normalized to the mean value of H2O2 generation by young vessels). The superoxide production of aortic rings was assessed with dihydroethidium (DHE) (D11347, Invitrogen, MA, USA) as previously described [35–37]. The frozen sections of aortic rings both without and with PVAT were cut into 10 µm thick sections and placed on plate. The samples were incubated at room temperature for 30 min with DHE (2 µM) and protected from light and the fluorescence intensity of the DHE staining was measured using a spectramax analyzer.

### Measurement of senescence

Senescence was measured using Senescence β-Galactosidase Activity Assay Kit (Fluorescence, Plate-Based). Heart or aortic single cell suspension were prepared using beads in senescence cell lysis buffer. Upon binding to β-gal, 4-Methylumbelliferyl β-D-galactopyranoside (4-MUG) is hydrolyzed to the fluorescent product 4-MU that can be measured at an excitation wavelength of 360 nm and an emission wavelength of 465 nm. Fluorescent intensities were measured using Spectramax M3.

### Statistical analysis

For the animal experiments all the values are represented as mean ± SEM. Statistical analysis of the data was performed by analysis of variance (two-way ANOVA) followed by Tukey’s post hoc test for multiple comparisons or t-test if appropriate as previously described. The analysis was conducted using GraphPad Prism 6 software. *p* < 0.05 was considered statistically significant.

## Results

### Chronic alcohol consumption impairs lipid metabolism and body weight gain.

To investigate the effects of alcohol on cardiovascular and metabolic aging, we utilized a chronic alcohol feeding model. Young Fisher F344BNF1 rats (3 months old) and aging rats (24–26 months old) were fed a 5% liquid alcohol diet or an isocaloric control diet for six months (Fig. 1A). The alcohol diet resulted in a significant increase in blood alcohol levels, reaching 136.6 ± 11. mg/dL in the young group and 144.2 ± 11.7 mg/dL in the aging group, respectively. We did not observe significant weight loss effects of alcohol in young animals, although it attenuated the time-dependent body weight gain seen in the pair-fed control group (Fig. 1C.). In the aging group, alcohol significantly reduced body weight by the end of the 6-month diet compared to animals on the control isocaloric diet (Fig. 1C).

Aging was associated with elevated serum LDL cholesterol levels. Although triglyceride levels were also higher with aging, the increase did not reach statistical significance. Chronic alcohol consumption significantly increased serum LDL cholesterol and triglyceride levels in both young and aging groups (Fig. 1D).

### Chronic alcohol consumption promotes cardiac mitochondrial dysfunction and reactive oxygen and nitrogen species (ROS/RNS) production.

**Fig. 2:**
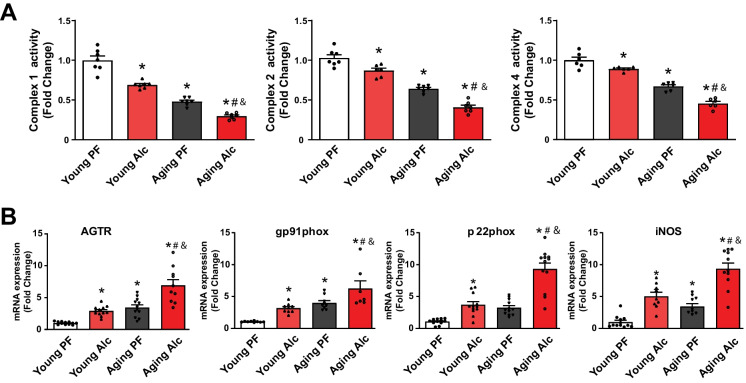
Effect of chronic alcohol diet in young and aging rats on myocardial mitochondrial complex activities and oxidative stress. **A **The effect of liquid 5% alcohol (Alc) or isocaloric control pair-fed (PF) diet for 6 months on myocardial mitochondrial complex I, II, and IV activities in young and aging rats is shown. Data represents the mean ± S.E.M, with *n* = 6–7 per group. *p < 0.05 vs. Young PF group, # *p* < 0.05 vs. Aging PF group, & *p* < 0.05 Young Alc vs. Aging Alc group. **B** The effect of liquid 5% alcohol (Alc) or isocaloric control diet for 6 months in young and aging rats on the myocardial transcripts of ROS-generating receptors/enzymes (AGTR, gp91phox, p22phox, and iNOS) is shown. Data represents the mean ± S.E.M, with *n* = 9–12 per group. *p < 0.05 vs. Young PF group, # *p* < 0.05 vs. Aging PF group, & *p* < 0.05 Young Alc vs. Aging Alc group

Given that mitochondrial dysfunction and oxidative stress are key pathological processes involved in cardiovascular aging, we investigated the impact of aging and chronic alcohol diet on these processes. Mitochondrial complex I, II, and IV activities were significantly reduced in the hearts of 30–32-month-old aging rats compared to 9-month-old young rats. Chronic alcohol consumption further significantly decreased mitochondrial complex I, II and IV activities in both young and aging rats (Fig. 2A).

In addition to impaired mitochondrial function, various receptors and enzymes may also contribute to the pathological generation of reactive oxygen and nitrogen species (ROS/RNS) during cardiovascular aging and alcoholic cardiomyopathy. To explore this, we investigated the effects of aging and alcohol on the myocardial expression of angiotensin II receptor (AGTR), NADPH oxidase isoforms gp91phox and p22phox, and inducible nitric oxide synthase (iNOS) (Fig. 2B). All four transcripts were elevated in both young and aging groups following the alcohol diet. However, the increases were more pronounced in the aging groups compared to the young groups (Fig. 2B).

**Fig. 3:**
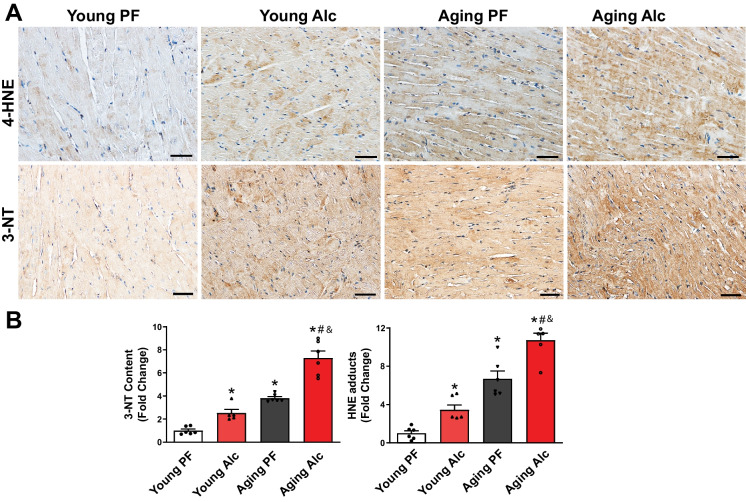
Effect of chronic alcohol diet in young and aging rats on myocardial oxidative/nitrative stress. **A** Representative immunostainings showing changes in 4-HNE (a stable marker of lipid peroxidation) and 3-NT (a marker of peroxynitrite formation and protein nitration) in the left ventricles of young and aging groups fed with 5% alcohol (Alc) or isocaloric pair-fed (PF) control liquid diets for 6 months. The scales represent 50 µm. **B** Bottom Panel: Quantitative determination of 4-HNE protein adducts and 3-NT content by ELISA. Data represents the mean ± S.E.M, with *n* = 5–6 per group. *p < 0.05 vs. Young PF group, # *p* < 0.05 vs. Aging PF group, & *p* < 0.05 Young Alc vs. Aging Alc group

Using histological staining of myocardial sections, we also evaluated the footprints of oxidative/nitrative stress by using two established markers: 4-hydroxynonenal (4-HNE) and 3-nitrotyrosine (3-NT) (Fig. 3). 4-HNE is a stable marker of lipid peroxidation, while 3-NT serves as a marker for peroxynitrite formation (a reactive nitrogen species), resulting in from the reaction of superoxide anion and nitric oxide [38]. More broadly, 3-NT is also an indicator of protein nitration and nitrative stress [38]. Representative immunostaining with antibodies against 4-HNE and 3-NT revealed increases in myocardial 4-HNE and 3-NT staining following alcohol consumption in both young and aging groups (Fig. 3A). However, the increase was significantly more pronounced in the aging group on the alcohol diet compared to the young animals. Additionally, we employed a more quantitative approach using ELISA to measure myocardial 4-HNE adducts and 3-NT content (Fig. 3B). Myocardial 4-HNE and 3-NT levels significantly increased in the young alcohol group compared to the pair-fed group. Aging alone also led to significant increases in these levels, which were further amplified by alcohol consumption (Fig. 3.B).

### Chronic alcohol consumption promotes myocardial inflammation.

**Fig. 4:**
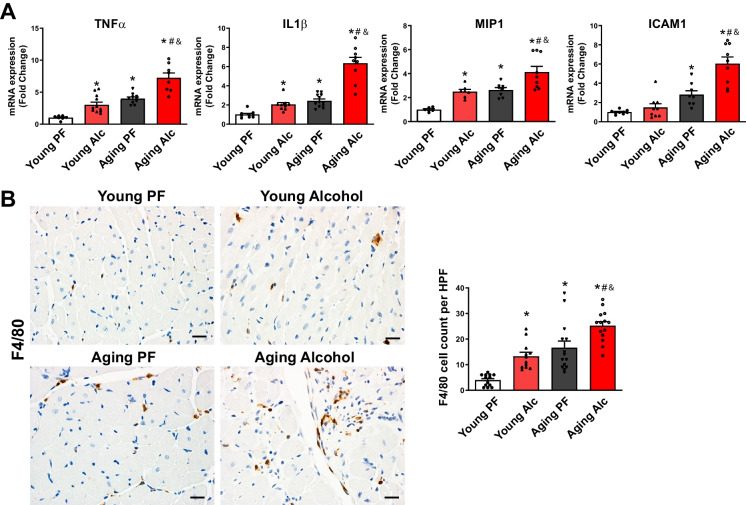
Effect of chronic alcohol diet in young and aging rats on myocardial inflammation. **A **Real-time PCR analyses reveal changes in the transcripts of cytokines/chemokines (TNFα, IL1β, MIP1α) and the adhesion molecule ICAM1 in the indicated groups following 5% alcohol (Alc) or isocaloric pair-fed (PF) control liquid diets for 6 months. Data represents the mean ± S.E.M, with *n* = 8–12 per group. *p < 0.05 vs. Young PF group, # *p* < 0.05 vs. Aging PF group, & *p* < 0.05 Young Alc vs. Aging Alc group. **B **Representative images of F4/80 staining (a macrophage marker) in left ventricular tissue sections taken at 400X magnification from indicated groups. The scales represent 20 µm. Quantification of F4/80 positive cells were performed from images in the high-power field (HPF). Data represents the mean ± S.E.M, with *n* = 12–14 per group. *p < 0.05 vs. Young PF group, # *p* < 0.05 vs. Aging PF group, & *p* < 0.05 Young Alc vs. Aging Alc group

Next, we investigated the role of inflammation in cardiovascular aging and the effects of chronic alcohol consumption (Fig. 4A, B). Transcript levels of the key inflammatory cytokine TNFα significantly increased in the young alcohol group compared to the pair-fed group. Aging alone also led to a significant increase, which was further amplified by alcohol consumption. Transcripts of cytokine IL1β increased significantly in the young alcohol group compared to the pair-fed group. Aging alone also led to an increase in IL1β transcripts, which was further amplified by alcohol consumption (Fig. 4A). Similarly, chemokine MIP1α transcript levels were elevated in both the young alcohol and aging groups compared to pair-fed young animals, with an even greater increase observed in the aging group consuming alcohol (Fig. 4A). However, transcripts of the adhesion molecule ICAM1 did not increase in the young alcohol group compared to the pair-fed group. Aging alone resulted in a significant increase in ICAM1 transcripts, which was further amplified by alcohol (Fig. 4A).

Myocardial immunostaining with F4/80, a marker of macrophage infiltration, revealed a significant increase in cell count in young rats on the alcohol diet compared to isocaloric controls. Aging alone caused a significant rise in cell count, which was further amplified by alcohol consumption (Fig. 4B).

### Chronic alcohol consumption promotes myocardial cell death and senescence.

**Fig. 5:**
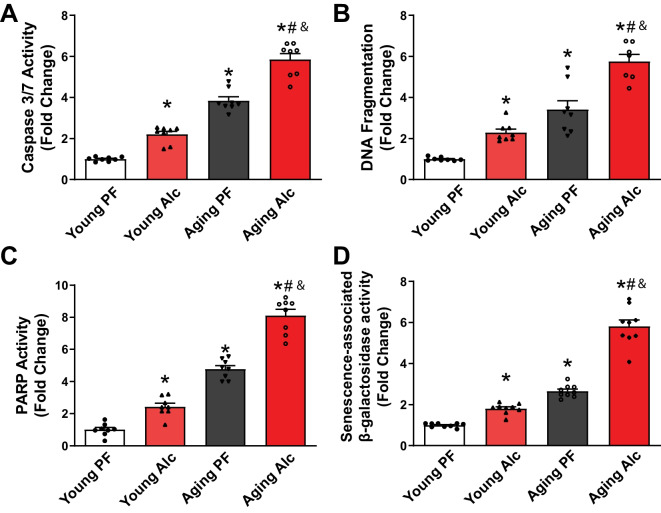
Effect of chronic alcohol diet in young and aging rats on myocardial cell death and senescence. **A**, **B **The effect of a 6-months liquid 5% alcohol (Alc) or isocaloric pair-fed (PF) diets on myocardial apoptotic cell death is shown in the indicated groups. **C **The effect on PARP-mediated cell death is also presented. Data represents the mean ± S.E.M, with *n* = 7–8 per group. *p < 0.05 vs. Young PF group, # *p* < 0.05 vs. Aging PF group, & *p* < 0.05 Young Alc vs. Aging Alc group. **D **The impact of a 6-month alcohol (Alc) or isocaloric control pair-fed (PF) diet on SA-β-galactosidase activity, a marker of cellular senescence, is shown in young and aging groups. Data represents the mean ± S.E.M, with *n* = 8 per group. *p < 0.05 vs. Young PF group, # *p* < 0.05 vs. Aging PF group, & *p* < 0.05 Young Alc vs. Aging Alc group

We next investigated the roles of apoptosis, poly (ADP-ribose) polymerase (PARP)-dependent cell death, and senescence in cardiovascular aging, along with the effects of chronic alcohol consumption (Fig. 5A-D). The activity of the early apoptotic marker caspase 3/7 was significantly increased in the young alcohol group compared to the pair-fed group. Aging alone also caused a significant increase in caspase 3/7 activity, which was further enhanced by alcohol consumption (Fig. 5A). Similarly, DNA fragmentation, another apoptotic marker, showed a significant rise in the young alcohol group compared to the pair-fed group. Aging alone led to an even greater increase in DNA fragmentation, which was further amplified by alcohol. PARP activity, a marker of cell death, also increased significantly in the young alcohol group compared to the pair-fed group. Aging alone resulted in a significant rise in PARP activity, which was further enhanced by alcohol consumption (Fig. 5C). We also measured the activity of the senescence marker SA-β-galactosidase (Fig. 5D). A small but significant increase was observed in the young alcohol group compared to the pair-fed group. Aging alone caused a greater increase in SA-β-galactosidase activity, which was further amplified by alcohol consumption (Fig. 5D).

### Chronic alcohol consumption promotes myocardial fibrosis in aging hearts.

**Fig. 6:**
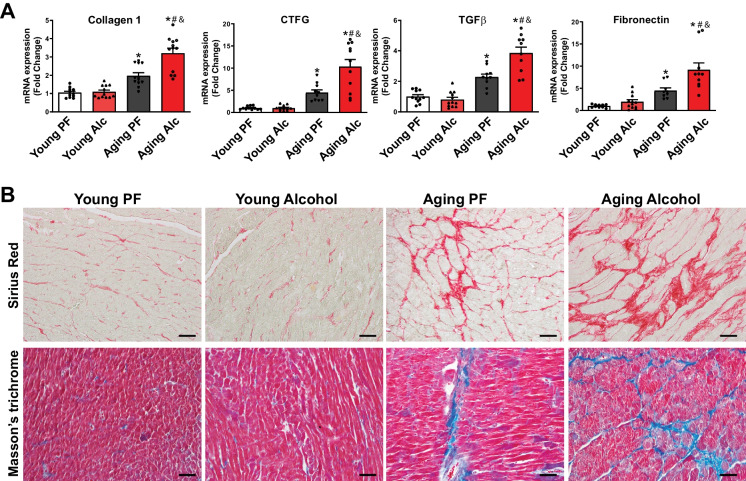
Effect of chronic alcohol diet in young and aging rats on myocardial fibrosis. The effect of a 6-months liquid diet with 5% alcohol (Alc) or isocaloric pair-fed (PF) control diets on left ventricular fibrosis is shown. **A **Real-time PCR analyses reveal changes in the transcripts of fibrosis markers: Collagen 1, CTGF, TGFβ, and fibronectin. Data represents the mean ± S.E.M, with *n* = 9–12 per group. *p < 0.05 vs. Young PF group, # *p* < 0.05 vs. Aging PF group, & *p* < 0.05 Young Alc vs. Aging Alc group. **B **Representative images of myocardial fibrosis staining with Sirius Red and Masson’s trichrome are shown for the indicated groups. Red or blue staining indicates myocardial fibrosis. The scales represent 50 µm

Given that myocardial fibrotic remodeling is a pathological consequence of chronic oxidative stress, inflammation, senescence, and cell death, we investigated the role of myocardial fibrosis in cardiovascular aging and the effects of chronic alcohol consumption (Fig. 6A-B). Real-time PCR analyses of fibrosis markers—Collagen 1, CTGF, TGFβ, and fibronectin—revealed a significant increase in the aging heart in response to chronic alcohol consumption, but not in the young group (Fig. 6A). These increases were further amplified by alcohol (Fig. 6A).

To further verify myocardial fibrosis, we stained the left ventricular tissue sections from each group with Sirius red and Masson’s trichrome, which are established methods for detecting fibrosis (Fig. 6B). Consistent with the real-time PCR data, there was no increase in fibrosis in the young groups, regardless of alcohol use, as shown in the representative images (Fig. 6B). In contrast, myocardial fibrosis (red or blue staining with Sirius Red or Masson’s trichrome, respectively) was evident in aging hearts and was significantly amplified by chronic alcohol consumption.

### Chronic alcohol consumption promotes left ventricular systolic dysfunction.

The hemodynamic effects of alcohol are complex, involving both cardiac and vascular influences that affect heart rate and loading conditions. Due to these complexities, conventional echocardiography may not reliably detect intrinsic heart rate and load-independent indexes of myocardial contractile function. Therefore, we utilized the invasive pressure–volume (PV) approach, considered the gold standard in hemodynamic measurements, to obtain accurate and comprehensive assessments of cardiac and vascular function in vivo.

**Fig. 7:**
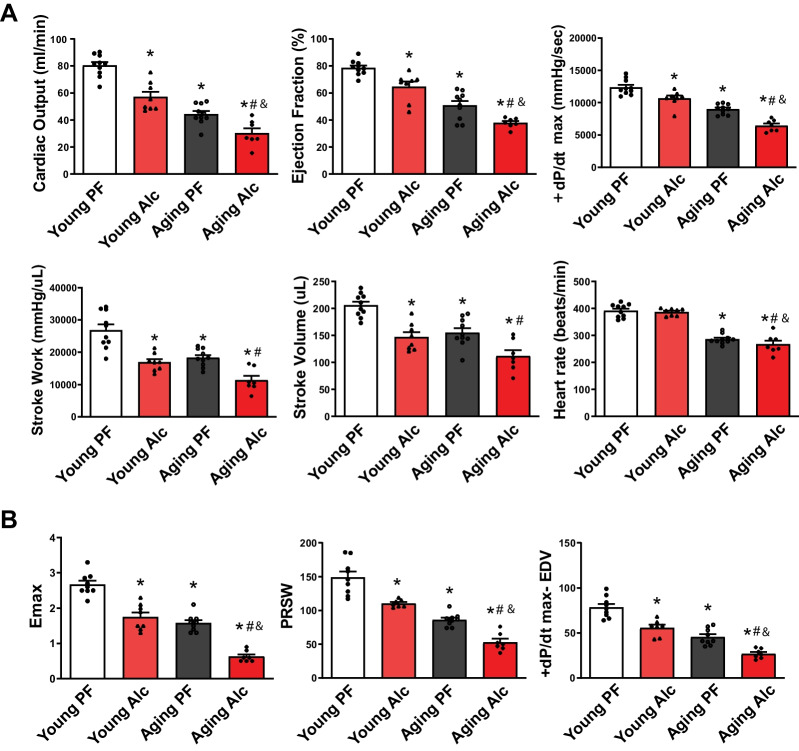
Effect of chronic alcohol diet in young and aging rats on left ventricular systolic function. The effect of a 6-months liquid diet with 5% (Alc) or isocaloric pair-fed (PF) control diet in young and aging rats on left ventricular systolic function indices is shown. **A **Load- and/or heart rate-dependent indices of left ventricular function, including cardiac output, ejection fraction, + dP/dt max (maximal rate of left ventricular pressure rise), stroke volume, stroke work, and heart rate. Data represents the mean ± S.E.M, with *n* = 7–10 per group. *p < 0.05 vs. Young PF group, # *p* < 0.05 vs. Aging PF group, & *p* < 0.05 Young Alc vs. Aging Alc group. **B **Load- and heart rate-independent indices of left ventricular contractile function, including Emax, PRSW, and + dP/dt-EDV, following a 6-month liquid diets with 5% alcohol (Alc) or isocaloric pair-fed (PF) control diets in young and aging rats. Data represents the mean ± S.E.M, with *n* = 7–9 per group. *p < 0.05 vs. Young PF group, # *p* < 0.05 vs. Aging PF group, & *p* < 0.05 Young Alc vs. Aging Alc group

Detailed hemodynamic measurements using the PV approach revealed that chronic alcohol consumption significantly decreased conventional markers of myocardial function in young animals compared to the isocaloric control group. These markers included ejection fraction, + dP/dt max, stroke work, stroke volume, and cardiac output, while the heart rate remained unaffected (Fig. 7A). Aging was also associated with a decline in these indices of left ventricular function, which was further exacerbated by alcohol consumption (Fig. 7A). Although aging led to a reduced heart rate, alcohol consumption did not further impact this parameter.

Through hemodynamic manipulations, specifically vena cava inferior occlusions, we also determined load- and heart rate-independent indices of myocardial contractile function. These included Emax (slope of the end-systolic pressure–volume relationship), preload recruitable stroke work (PRSW, the relationship between ventricular stroke work and end-diastolic volume), and + dP/dt-EDV (the relationship between + dP/dt and end-diastolic volume) (Fig. 7B). This analysis revealed that these intrinsic indices of myocardial contractile function were impaired by alcohol in the young group and by aging. The decrease in these indices was further exacerbated by alcohol consumption in the aging group (Fig. 7B).

### Chronic alcohol consumption promotes left ventricular diastolic dysfunction in aging hearts.

**Fig. 8:**
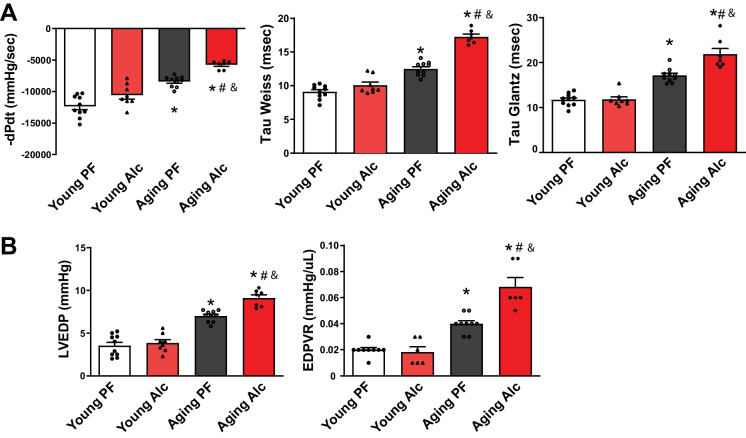
Effect of chronic alcohol diet in young and aging rats on left ventricular diastolic function. The effect of a 6-months liquid diet with 5% alcohol (Alc) or isocaloric pair-fed (PF) control diets in young and aging rats on left ventricular diastolic function indices is shown. **A **Changes in -dP/dt, Tau Weiss, and Tau Glantz following the indicated dietary interventions. Data represents the mean ± S.E.M, with *n* = 7–10 per group. *p < 0.05 vs. Young PF group, # *p* < 0.05 vs. Aging PF group, & *p* < 0.05 Young Alc vs. Aging Alc group. **B **The effect of a 6-months liquid diet with 5% alcohol (Alc) or isocaloric pair-fed (PF) control diets in young and aging groups on left ventricular end-diastolic pressure (LVEDP) and end-diastolic pressure–volume relationship (EDPVR), the latter being an index of myocardial stiffness/fibrosis. Data represents the mean ± S.E.M, with *n* = 6–10 per group. *p < 0.05 vs. Young PF group, # *p* < 0.05 vs. Aging PF group, & *p* < 0.05 Young Alc vs. Aging Alc group

Next, we evaluated indices of left ventricular diastolic function, including -dP/dt, the left ventricular diastolic time constant Tau (Weiss and Glantz), left ventricular end-diastolic pressure (LVEDP), and the slope of the end-diastolic pressure–volume relationship (EDPVR) (Fig. 8A, B). In young animals, alcohol consumption did not affect diastolic functional parameters, contrasting with its impact on systolic function. However, in aging animals, diastolic functional parameters were already impaired, and chronic alcohol consumption further exacerbated these impairments (Fig. 8A, B).

### Chronic alcohol consumption increases vascular oxidative/nitrative stress, apoptosis, and senescence.

**Fig. 9:**
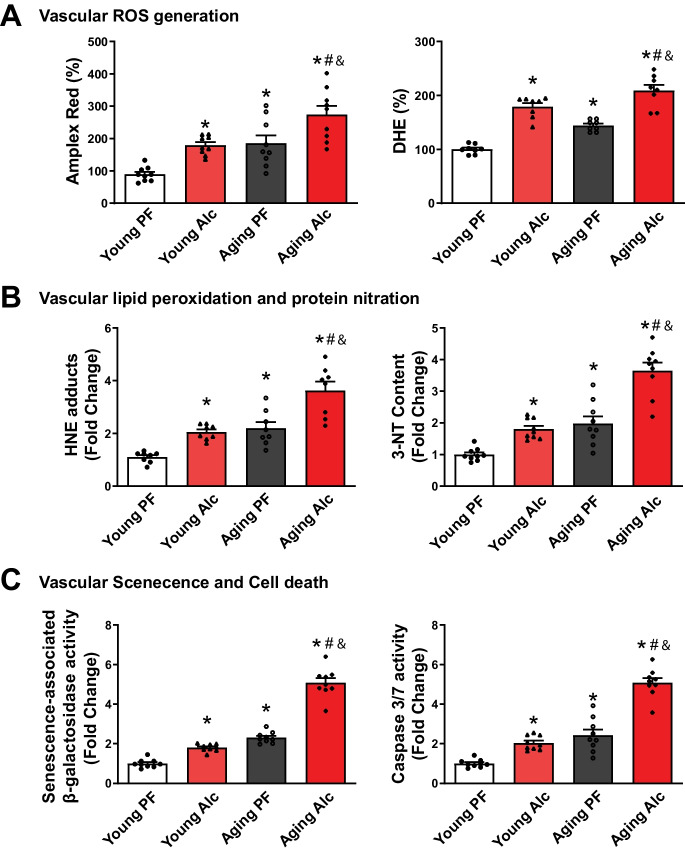
Effect of chronic alcohol diet in young and aging rats on vascular oxidative/nitrative stress, cell death and senescence. The effect of a 6-months liquid diet with 5% alcohol (Alc) or isocaloric pair-fed (PF) control diets in young and aging rats on vascular oxidative/nitrative stress, cell death, and senescence markers is shown. **A **Vascular ROS generation was determined from aortic lysates using Amplex Red for hydrogen peroxide and DHE for superoxide in the indicated groups. Data represents the mean ± S.E.M, with *n* = 8–9 per group. *p < 0.05 vs. Young PF group, # *p* < 0.05 vs. Aging PF group, & *p* < 0.05 Young Alc vs. Aging Alc group. **B **Oxidative/nitrative stress markers 4-HNE adducts, and 3-NT content were measured from aortic lysates of the indicated groups. Data represents the mean ± S.E.M, with *n* = 9 per group. *p < 0.05 vs. Young PF group, # *p* < 0.05 vs. Aging PF group, & *p* < 0.05 Young Alc vs. Aging Alc group. **C **Measurements of SA-β-galactosidase (a marker of senescence) and caspase 3/7 (a marker of apoptotic cell death) activities are shown for the indicated groups. Data represents the mean ± S.E.M, with *n* = 9 per group. *p < 0.05 vs. Young PF group, # *p* < 0.05 vs. Aging PF group, & *p* < 0.05 Young Alc vs. Aging Alc group

In addition to its effects on the heart, aging and alcohol also significantly impact the vasculature. Therefore, we evaluated the effects of aging and alcohol on vascular reactive oxygen species (ROS) generation (measured by H_2_O_2_ generation using Amplex Red and superoxide generation using dihydroethidium (DHE) assays), lipid peroxidation (using 4-HNE ELISA), nitrative stress (using 3-NT ELISA), apoptosis (using caspase 3/7 activity assay), and senescence (using β-galactosidase activity assay) using aortic lysates (Fig. 9A-C). In both young and aging animals, alcohol consumption significantly increased vascular ROS generation, lipid peroxidation, protein nitration, apoptosis, and senescence (Fig. 9A-C).

### Chronic alcohol consumption promotes endothelial dysfunction, increases total peripheral resistance, ventricular-arterial uncoupling and decreases cardiac efficiency

**Fig. 10:**
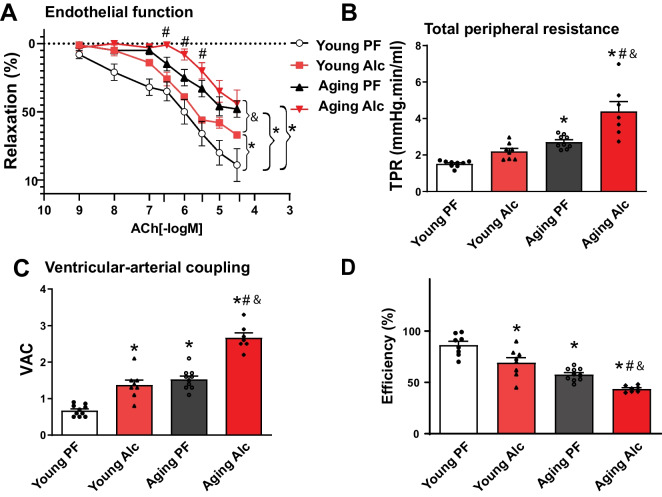
Effect of chronic alcohol diet in young and aging rats on endothelial function, total peripheral resistance, ventricular-arterial coupling, and efficiency. The effect of a 6-months liquid diet with 5% alcohol (Alc) or isocaloric pair-fed (PF) control diets in young and aging rats is shown on: **A **Endothelial-dependent relaxation response of aortic rings to Acetylcholine (ACh). **B **Total peripheral resistance (TPR). **C **Ventricular-arterial coupling (VAC). **D **Cardiac mechanical efficiency. Data represents the mean ± S.E.M, with *n* = 7–10 per group. *p < 0.05 vs. Young PF group, # *p* < 0.05 vs. Aging PF group, & *p* < 0.05 Young Alc vs. Aging Alc group

Chronic vascular oxidative stress, cell death, and senescence can impair vascular function and remodeling, leading to endothelial dysfunction, increased total peripheral resistance (TPR), and decreased reserve capacity of the vascular system. These changes may also reduce the efficiency of ventriculo-arterial coupling, thus diminishing the overall reserve capacity of the cardiovascular system. To investigate the effects of aging and chronic alcohol consumption on these parameters, we evaluated endothelium-dependent relaxation of isolated aortic rings, TPR, ventriculo-arterial coupling ration, and mechanical efficiency in vivo using the PV approach (Fig. 10A-D).

Chronic alcohol intake in both young and aging rats resulted in decreased endothelium-dependent vasorelaxation of isolated aortic rings, indicating vascular dysfunction (Fig. 10A). Aging was associated with increased TPR, which was further exacerbated by alcohol consumption (Fig. 10B). Additionally, alcohol promoted ventriculo-arterial uncoupling and decreased cardiac efficiency, particularly in aging animals, where these variables were already impaired (Fig. 10C, D).

## Discussion

Genetic studies indicate that alcohol consumption may shorten lifespan [16–21]. Coupled with rising alcohol use among the aging population and their heightened risk of cardiovascular disease [22], this underscores the urgent need for a deeper understanding of alcohol’s effects on both normal and aging cardiovascular system. Despite these concerns, there are very few studies investigating the impact of chronic alcohol consumption on cardiovascular aging [39].

Our study, using a well-established rat model that closely resembles human cardiovascular aging, reveals that prolonged heavy drinking accelerates the primary aging processes in the heart and blood vessels. This acceleration occurs through mitochondrial dysfunction, increased oxidative and nitrative stress, inflammation, cell death, senescence, and dysregulation of lipid metabolism. These changes contribute to the development and progression of cardiovascular remodeling and dysfunction. Moreover, alcohol aggravates the existing uncoupling of the vascular and cardiac systems associated with aging, further diminishing the heart’s mechanical efficiency, and leading to a reduced cardiovascular reserve capacity.

Excessive chronic alcohol consumption can lead to the development of nonischemic dilated cardiomyopathy with myocardial fibrosis, known as alcoholic (more recently termed alcohol-associated) cardiomyopathy. This condition is characterized by the dilation of the left ventricle and impaired cardiac function [26, 40, 41]. Additionally, excessive alcohol intake impairs vascular function and structure both directly and indirectly. The detrimental effects of alcohol on the cardiovascular system are complex, involving mitochondrial dysfunction, oxidative and nitrative stress, chronic inflammation, parenchymal cell death, impaired protein synthesis and lipid metabolism, autonomic nervous system dysfunction, activation of the renin-angiotensin and sympathetic nervous systems, and hormonal imbalances, among other factors [26, 28, 40, 42, 43]. These pathological processes ultimately lead to cardiovascular dysfunction, pathological remodeling of the heart and vascular system, decreased cardiovascular reserve capacity, and eventually heart failure when the reserve capacity is exhausted.

Ethanol undergoes enzymatic transformation primarily through alcohol dehydrogenase (ADH) or Cytochrome P450 2E (CYP2E1), initially converting it into acetaldehyde. This acetaldehyde is subsequently transported into mitochondria, where aldehyde dehydrogenase metabolizes it into acetate and further into acetyl-CoA. This metabolic pathway results in the production of reactive oxygen species (ROS) such as superoxide, which leads to significant oxidative stress [44, 45]. In addition to mitochondrial impairment, various enzymes including NADPH oxidases and uncoupled inducible nitric oxide synthase (iNOS) can also contribute to ROS generation under pathological conditions. Superoxide can react with nitric oxide (NO) in a diffusion-limited manner, forming peroxynitrite, a reactive nitrogen species (RNS) [38]. Both ROS and RNS damage cellular components such as contractile proteins, enzymes, and mitochondria through oxidation and nitration processes, exacerbating stress signaling, cellular dysfunction, and ultimately leading to cell death in cardiomyocytes and endothelial cells [38].

Consistent with the importance of mitochondrial dysfunction and increased ROS/RNS generation in the pathology of alcohol-induced cardiomyopathy and vascular dysfunction we found decreased myocardial mitochondrial function (complex 1, 2 and 4 activities) and increased expression of angiotensin II receptor and its downstream effector ROS generating enzyme NADPH oxidase (gp91 and p22phox isoforms) in young rats exposed to chronic liquid alcohol diet. Similarly, there was an increased myocardial expression of the inducible nitric oxide synthase (iNOS) (Fig. 2), which could be a significant source of nitric oxide under pathological conditions and when uncoupled can also generate ROS. These alterations, akin to those observed in isolated vessels, were paralleled by elevated levels of lipid peroxidation and protein nitration in the cardiovascular system of young rats exposed to alcohol (Figs. 3 and 9).

ROS/RNS through the activation of various stress signaling pathways and activation of transcription factors can also promote inflammation, cell death and senescence in the cardiovascular system. In line with this we found that chronic alcohol consumption in young rats enhanced myocardial macrophage infiltration and expression of various pro-inflammatory cytokines/chemokines (TNFα, ILβ, and MIP-1) (Fig. 4.), which was also accompanied by increased cell death (apoptotic and PARP dependent) and senescence (Fig. 5), but not myocardial fibrosis (Fig. 6). The predominant oxidative and nitrative stress driven pathology accompanied by modest myocardial inflammation, elevated serum triglyceride levels and absence of myocardial fibrosis in young rats on a chronic alcohol diet, and the associated impaired systolic function and endothelium-dependent vasorelaxation aligns well with previously reported findings in mouse models [28].

Consistent with the literature [3, 5, 46–48] we observed impaired mitochondrial function, heightened oxidative and nitrative stress, inflammation, cell death, senescence, and myocardial fibrosis in the hearts and/or vasculature of aging animals (Figs. 1, 2, 3, 4, 5, 6). Additionally, impaired lipid metabolism was evident, reflected by elevated serum LDL cholesterol levels. Chronic alcohol consumption in aging rats further aggravated these pathological processes (Figs. 1, 2, 3, 4, 5, 6).

Recent preclinical and clinical studies have highlighted a potential link between the development of metabolic dysfunction-associated steatohepatitis (MASH; formerly termed non-alcoholic steatohepatitis (NASH)) and cardiac dysfunction, including aging-related cardiac impairment [5, 12, 49]. Given that alcohol consumption is a known driver of alcohol related steatohepatitis (ASH) and that ALD worsens with aging, it is reasonable to hypothesize that ALD in aging may also contribute indirectly to cardiac dysfunction.

Research into alcohol-associated cardiomyopathy extensively explores cardiac performance using invasive, noninvasive, and in vitro methods [26, 39, 40, 43, 44, 50]. Yet, debates persist regarding the overall impact of ethanol consumption on cardiovascular function and disease, as it can be dose- and time-dependent, affecting not only the heart but also the vasculature, influencing either vasoconstriction or vasodilation [26]. Most studies investigating alcohol-induced cardiovascular function have relied on the use of conventional echocardiography. However, conventional echocardiographic measurements are dependent on loading conditions and heart rate and are often unreliable in conditions with significant vascular alterations, which occur during both acute [51] and chronic [28] alcohol consumption. Similar changes also occur during cardiovascular aging [52]. Therefore, to reliably investigate the effects of ethanol consumption on cardiovascular performance, a comprehensive analysis of cardiac and vascular function is required.

Thus, to determine the cardiovascular consequences of chronic alcohol consumption we utilized invasive hemodynamic examination and pressure–volume (P–V) analysis [31, 52, 53], a gold standard in complex hemodynamic measurements. P–V analysis enables the measurement of pre- and afterload, as well as heart rate-independent systolic contractility parameters, specific diastolic function, cardiac stiffness, and vascular parameters (elastance, total peripheral resistance, etc.). It also allows calculations of important mechanoenergetic parameters, such as ventriculo-arterial coupling (VAC) and mechanical efficiency. In addition to the P–V approach, we also investigated the vascular function in isolated aortic rings by evaluating the endothelial-dependent vasorelaxant response to acetylcholine.

We observed a significant reduction in systolic indices, including cardiac output, ejection fraction, + dP/dtmax, stroke work, Ees, PRSW, and + dP/dtmax-EDV, indicating left ventricular (LV) contractile dysfunction in both young and aging rats subjected to chronic ethanol consumption (Fig. 7). These detrimental effects of alcohol on systolic cardiac performance were notably more pronounced in aging animals. Aging animals in our study on a pair-fed diet exhibited characteristic functional changes (Fig. 7) similar to those previously described [5, 30, 34].

In addition to prominent systolic dysfunction, we noted a significant worsening of diastolic LV relaxation, evidenced by increased Taus and decreased dP/dtmin, in aging animals (Fig. 8). This was accompanied by increased LV stiffness, as measured by LVEDP and the slope of EDPVR. These pathological changes were further exacerbated by alcohol in aging rats and were consistent with the increased fibrosis observed in these groups.

Regarding vascular indices, we observed an increase in total peripheral resistance (TPR) in aging animals, which was further exacerbated by chronic alcohol consumption (Fig. 10). Additionally, chronic alcohol consumption impaired endothelial-dependent vascular function in isolated aortic rings both in young and aging rats (Fig. 10). Our functional data align with in vitro findings, indicating that significant myocardial oxidative/nitrative stress and inflammation, combined with mitochondrial dysfunction, impaired lipid metabolism, increased cell death and senescence, leads to an energetic crisis. This crisis combined with remodeling (fibrosis) results in the observed impairments in cardiac contractility and diastolic relaxation.

We also employed P–V analysis to assess the mechanoenergetic parameters, such as ventriculo-arterial coupling (VAC) and efficiency. Ventriculo-arterial coupling is the ratio of Ees (end-systolic elastance) to Ea (arterial elastance) [54, 55]. Venticulo-arterial coupling is a critical determinant of cardiovascular health, reflecting the efficiency of the heart and vascular system working together [54, 55]. Disruption in this balance, observed in aging, hypertension, and heart failure, is linked to poor cardiovascular outcomes, and serves as a significant predictor of morbidity and mortality [54–56]. Mechanoenergetic efficiency (related to Ees, and the P–V area and calculated as the ratio of stroke work (SW) and pressure–volume area (PVA)) is an important measure of cardiac performance, reflecting how effectively the heart converts metabolic energy into mechanical work [57, 58]. In cardiovascular diseases such as heart failure, hypertension, ischemic heart disease, aging, and diabetes, reduced mechanoenergetic efficiency is common and is associated with increased morbidity and mortality [57, 59].

Chronic alcohol consumption both in young and aging animals significantly increased the ventriculo-arterial coupling ratio, indicating a mismatch (uncoupling) between the left ventricle’s contractility and the arterial system’s load, leading to inefficient stroke volume transmission, increased cardiac workload, reduced cardiac efficiency, and a higher risk of cardiovascular events. Consistently with arterio-ventricular uncoupling, we also found that chronic alcohol consumption in both young and aging rats reduced cardiac efficiency. This reduction suggests a decrease in metabolic efficiency, likely caused by mitochondrial disturbances and oxidative/nitrative stress observed in the hearts of the experimental animals. Understanding and assessing these parameters in subjects with alcohol use disorder can provide valuable insights into patient risk and guide therapeutic strategies to optimize cardiovascular function and improve patient outcomes.

Although the available literature is limited, evidence suggests that in the early stages of alcohol-associated cardiomyopathy, where cardiomyocyte death and fibrotic remodeling have not yet occurred, myocardial dysfunction may be reversible [60]. In such cases, complete alcohol abstinence can lead to significant improvements in cardiac function. However, in advanced stages, prolonged alcohol consumption results in irreversible structural damage, significantly limiting recovery even after cessation [60]. Regular exercise improves ventricular-arterial coupling in the elderly by reducing arterial stiffness and improving ventricular contractility, leading to more efficient energy transfer between the heart and arteries [61]. Given these benefits, it is plausible that combining alcohol abstinence with regular exercise could positively impact cardiovascular function in alcohol-associated cardiomyopathy by mitigating vascular stiffness, improving cardiac efficiency, and potentially slowing disease progression.

**Fig. 11:**
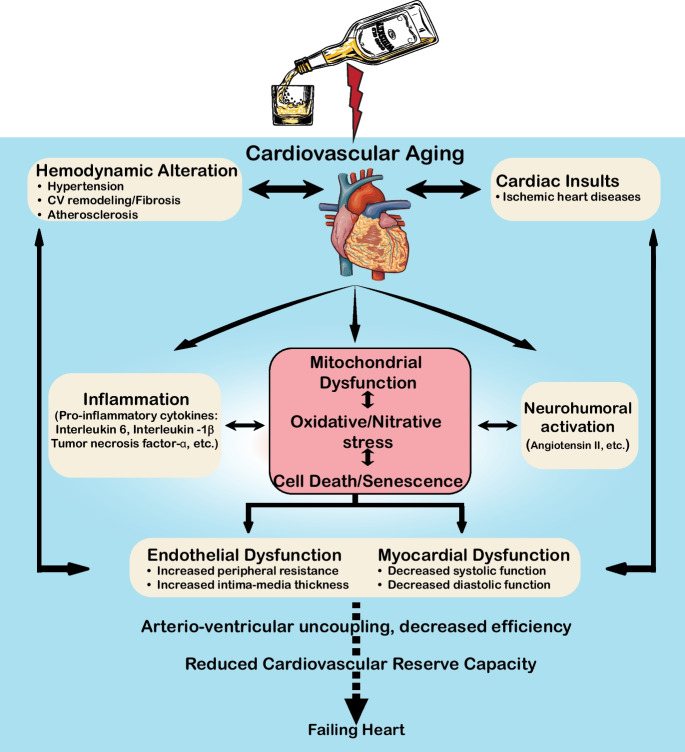
Effects of alcohol on cardiovascular aging and reserve capacity. A schematic illustration depicting the mechanisms by which alcohol promotes cardiovascular aging and reduces cardiovascular reserve capacity

Collectively, our findings demonstrate that chronic alcohol consumption accelerates cardiovascular aging (Fig. 11). Additionally, we show that long-term drinking impairs the efficiency of the heart and vascular system, diminishing the heart’s ability to convert metabolic energy into mechanical work. This leads to a decreased cardiovascular reserve capacity and an increased risk of cardiovascular disease.

## Supplementary Information

Below is the link to the electronic supplementary material.

ESM 1 (DOCX 15.4 KB)
