# Supplementary material for: Chronic alcohol consumption accelerates cardiovascular aging and decreases cardiovascular reserve capacity
Source: GeroScience. 2025 Mar 20;47(4):5881–901. doi: 10.1007/s11357-025-01613-w (PMC12397455; doi:10.1007/s11357-025-01613-w)
Supplement: Supplementary file 1 — (DOCX 15.4 KB) [file 11357_2025_1613_MOESM1_ESM.docx]

| **Custom designed** | **Forward sequence** | **Reverse sequence** |
| --- | --- | --- |
| gp91phox | GATCTTCTTCATCGGCCTTGC | CCCACGATCCATTTCCAAG |
| p22phox | TTACTGCTGTCCGTGCCTGC | CTTGATGGTGCCTCCAACCTG |
| iNOS | GAGAGATCCGGTTCACAGTCTTG | GCAGACAACCTTGGTGTTGAAG |
| TNFa | TCCCTCAGGGGTGTCCTTAG | GTCGTAGCAAACCACCAAGC |
| IL1b | TAATGCCTTCCCCAGGACATG | TGCCACGGTTTTCTTATGGC |
| MIP1 | CTCCTATGGACGGCAAATTCC | ATCTGCCGGTTTCTCTTGGTC |
| ICAM1 | CCAAGGAGATCACATTCACGG | AAGATCGAAAGTCCGGAGCTG |
| Col1a1 | GGAGAGAGCATGACCGATGG | AAGTTCCGGTGTGACTCGTG |
| CTGF | CTGACCTAGAGGAAAACATT | AGAAAGCTCAAACTTGACAG |
| Fibronectin | AAGACAGATGAGCTTCCCCA | TGGTTCGCCTAAAGCCATGT |
| Myh 6 | CCCTATAAGTGGCTGCCAGTGT | GTACTGATAGGCGTTGTCAGAGATG |
| TGF-beta1 | GCTGCTGACCCCCACTGAT | GCCACTGCCGGACAACTC |
| TNF-alpha | TCCCTCAGGGGTGTCCTTAG | GTCGTAGCAAACCACCAAGC |
| **Commercially available from Qiagen** | | **Catalog number** |
| Beta actin | | QT00193473 |
| Beta-2-microglobulin | | QT00176295 |
| AGTR1 | | QT00379295 |

**Supplemental Table1: Primers used in this study.**
